# Vagus nerve stimulation alleviated cerebral ischemia and reperfusion injury in rats by inhibiting pyroptosis via α7 nicotinic acetylcholine receptor

**DOI:** 10.1038/s41420-022-00852-6

**Published:** 2022-02-08

**Authors:** Hao Tang, Jiani Li, Qinxiang Zhou, Sheng Li, Chenchen Xie, Lingchuan Niu, Jingxi Ma, Changqing Li

**Affiliations:** 1 Department of Neurology, The Second Affiliated Hospital of Chongqing Medical University, Chongqing, China; 2 Chongqing Key Laboratory of Neurology, The First Affiliated Hospital of Chongqing Medical University, Chongqing, China; 3 Department of Rehabilitation Medicine, The First Affiliated Hospital of Chongqing Medical University, Chongqing, China; 4 Department of Neurology, Chongqing General Hospital, University of Chinese Academy of Sciences, Chongqing, China; 5 Chongqing Key Laboratory of Neurodegenerative Disease, Chongqing, China

**Keywords:** Cell death in the nervous system, Cellular neuroscience

## Abstract

Cumulative evidence suggests that pyroptosis, a new sort of programmed cell death, is closely related to cerebral ischemia/reperfusion (I/R) injury. Our previous studies have testified that vagus nerve stimulation (VNS) was involved in many different neuroprotective and neuroplasticity pathways via α7 nicotinic acetylcholine receptor (α7nAchR), a vital node of the cholinergic anti-inflammatory pathway during cerebral I/R injury. We aimed to determine the neuroprotective effects of VNS through α7nAchR-mediated inhibition of pyroptosis. Focal cerebral ischemic stroke rat models were obtained by middle cerebral artery occlusion for 120 min. Expression of the NLRP3 inflammasome was evaluated using western blotting and immunofluorescence (IF) staining. The neurological deficit score, infarct volume, TUNEL staining findings, transmission electron microscopy findings, and expression of inflammatory cytokines were assessed 3 days after I/R injury. Our findings suggested that the protein expression levels of NLRP3, GSDMD-N, cleaved caspase-1, and ASC gradually increased until they peaked on day 3 after I/R injury. VNS inhibited the expression of pyroptosis-related molecules and decreased the number of pyroptotic cells and membrane pores. Administration of α7nAchR-antagonist and agonist helped in further assessment of the role of α7nAchR in pyroptosis. α7nAchR-agonist mimicked VNS’s neuroprotective effects on the improvement of neurological deficits, the reduction of infarct volumes, and the inhibition of neuronal pyroptosis after cerebral I/R injury. Conversely, the neuroprotection provided by VNS could be reversed by the administration of α7nAchR-antagonist. In conclusion, VNS-induced neuroprotection via inhibition of neuronal pyroptosis was α7nAchR-dependent, highlighting the pivotal role of α7nAChR in suppressing cellular pyroptosis and neuroinflammation. These findings may allow a better understanding of treatment principles for cerebral I/R injury.

## Introduction

Currently, ischemic stroke is one of the well-known leading causes of death and long-term disability globally [1–3]. Although considerable efforts have been made to search for better treatment modalities for ischemic stroke, remarkably few methods are considered sufficiently effective due to their complex pathophysiological mechanism. Recently, several studies have focused on neuroinflammation since it plays a crucial role in the pathogenesis of ischemic stroke [4].

Pyroptosis, also known as cellular inflammatory necrosis, is a newly discovered type of programmed cell death characterized by cellular swelling and lysis, plasma membrane rupture, and release of pro-inflammatory cytokines, which ultimately amplify the inflammatory response [5–7]. The most distinctive biochemical feature of pyroptosis is that its occurrence and development depend on the activation of inflammasomes [8]. NLRP3 inflammasome is the most researched and plays a pivotal role in inducing pyroptosis. Many endogenous danger signals produced by cerebral ischemia/reperfusion (I/R) injury activate NLRP3 inflammasome and convert pro-caspase 1 into cleaved caspase-1 (Cl-Caspase-1). Cl-Caspase-1 causes gasdermin D (GSDMD) to be cleaved, allowing the N-terminal fragment (GSDMD-N) to bind to phosphatidylserine and finally rupture the cell membrane, releasing inflammatory cytokines [2]. Furthermore, activated caspase-1 transforms the precursors of interleukin (IL)−1β and IL-18 to cleaved IL-1β and IL-18. These active forms are discharged into the environment, where they recruit inflammatory cells for aggregation and increase the inflammatory response.

VNS is a safe and effective treatment that was first used to manage intractable epilepsy [9–11]. Several studies have reported that VNS can provide neuroprotective effects by activating the cholinergic anti-inflammatory pathway (CAP) after I/R injury [12, 13]. CAP works by transmitting the associated inflammatory signals from the vagus nerve (VN) fibers to the nucleus tractus solitarius, which projects to the dorsal nucleus of the VN. In turn, the dorsal nucleus activates the efferent branch of VN to release acetylcholine, which binds to the α7 nicotinic acetylcholine receptor (α7nAchR) for its anti-inflammatory function [14, 15]. α7nAchR, the most crucial molecule in CAP, can be activated and upregulated by VNS-induced endogenous acetylcholine [16]. Reportedly, α7nAchR-mediated CAP is closely related to many diseases in the central nervous system (CNS). Results of our previous study suggested that α7nAchR mediates VNS-induced neuroprotection via inhibition of apoptosis and modulation of neuroinflammation [17, 18]. Besides in CNS, VNS also suppresses inflammation in ARDS by inhibiting inflammatory pyroptosis and promoting M1 to M2 macrophage transformation through α7nAchR-mediated CAP [14]. Nevertheless, to the best of our knowledge, no research has analyzed the correlation between α7nAchR-mediated CAP and pyroptosis in ischemic stroke.

Therefore, the present study aimed to verify our assumption that α7nAchR mediates VNS-induced neuroprotection by inhibiting pyroptosis in cerebral I/R injury.

## Results

### Physiological parameters

The physiological parameters, which included the mean values for blood pressure, HR, and blood gases, remained within the normal limits in all groups (Table 1). Throughout the experiment, no remarkable changes in the parameters following VNS were observed, which is consistent with our previous research [18].

**Table 1:** The physiological parameters during the experiment (all data are shown as the mean ± SD).

Group	Mean blood pressure(mmHg)	Heat rate(bp/min)	PH	PCO2	PO2
Sham	85 ± 5.6	366 ± 8	7.39 ± 0.02	46.7 ± 1.1	112.4 ± 9.5
MCAO	84 ± 4.3	368 ± 10	7.38 ± 0.01	45.9 ± 0.9	110.8 ± 10.2
MCAO + VNS	86 ± 7.1	365 ± 9	7.38 ± 0.01	46.3 ± 1.2	114.5 ± 11.4
MCAO + VNS + MLA	84 ± 6.9	366 ± 11	7.40 ± 0.02	47.1 ± 0.8	110.5 ± 12.3
MCAO + VNS + Vehicle	85 ± 6.4	364 ± 12	7.39 ± 0.03	46.5 ± 1.0	109.7 ± 8.9
MCAO + Vehicle	87 ± 5.2	367 ± 10	7.37 ± 0.01	46.4 ± 1.0	112.9 ± 10.1
MCAO + GTS-21	85 ± 4.8	365 ± 11	7.41 ± 0.02	45.8 ± 1.3	111.3 ± 11.8

### Time course of NLRP3 inflammasome expression after MCAO in cerebral infarction

**Fig. 1:**
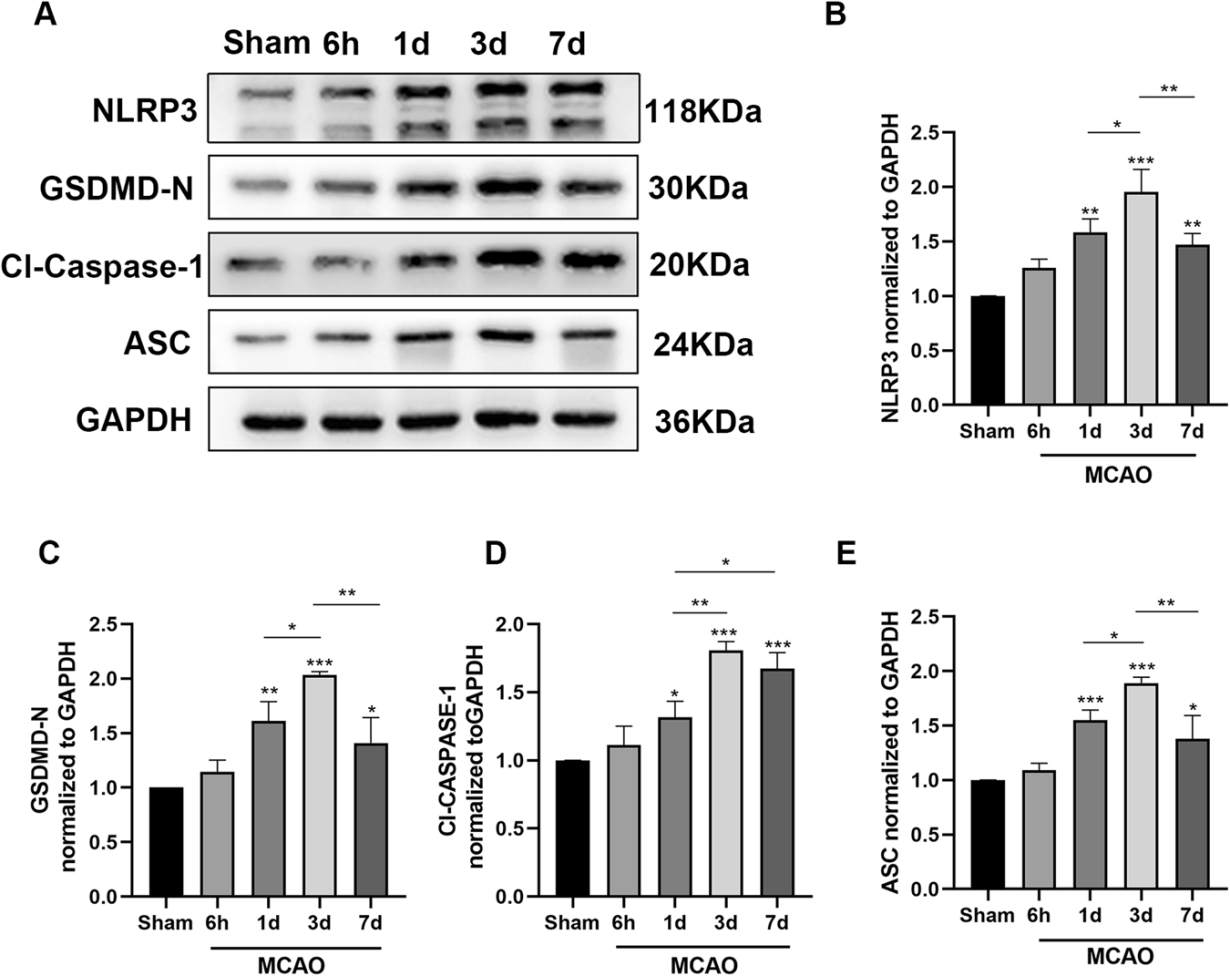
NLRP3 inflammasome expression was increased after cerebral ischemia/reperfusion injury. **A**–**E** Representative western blotting images and analysis of NLRP3, GSDMD-N, cleaved caspase 1 (Cl-Caspase-1), and ASC levels (*n* = 4). Cerebral ischemia/reperfusion injury substantially increased the levels of NLRP3, GSDMD-N, Cl-Caspase-1, and ASC, with the greatest level seen on day 3 following reperfusion. *P < 0.05, **P < 0.01, ***P < 0.001 versus the sham group.

To explore the time course of NLRP3 inflammasome expression after the cerebral infarction, its protein levels were recorded at different time points after 120 min following the MCAO injury. The expression levels of NLRP3, ASC, Cl-Caspase-1, and GSDMD-N at different time points after MCAO are depicted in Fig. 1. Western blotting results suggested that the protein expression level of NLRP3 inflammasome gradually increased until it reached a peak on day 3 after MCAO and reperfusion, followed by a gradual decrease. When compared with the sham group, NLRP3 expression (consistent with ASC and GSDMD-N expression) increased sharply on day 1 after I/R injury, remained at the highest level on day 3 after reperfusion, and gradually decreased after that (Fig. 1A–C, E). However, Cl-Caspase-1 exhibited only a slight increase in its expression on day 1 after reperfusion compared with the sham group. Subsequently, it rapidly increased and reached its peak on day 3 after cerebral I/R injury, gradually decreased after that, and maintained a high expression level on day 7 (Fig. 1A, D). In conclusion, when compared to the sham group, the contents of the NLRP3 inflammasome, which contains NLRP3, GSDMD-N, Cl-Caspase-1, and ASC, exhibited a rising trend at various time points following cerebral I/R injury, with the peak expression level observed on day 3 after reperfusion.

### VNS attenuated NLRP3 inflammasome expression after MCAO

**Fig. 2:**
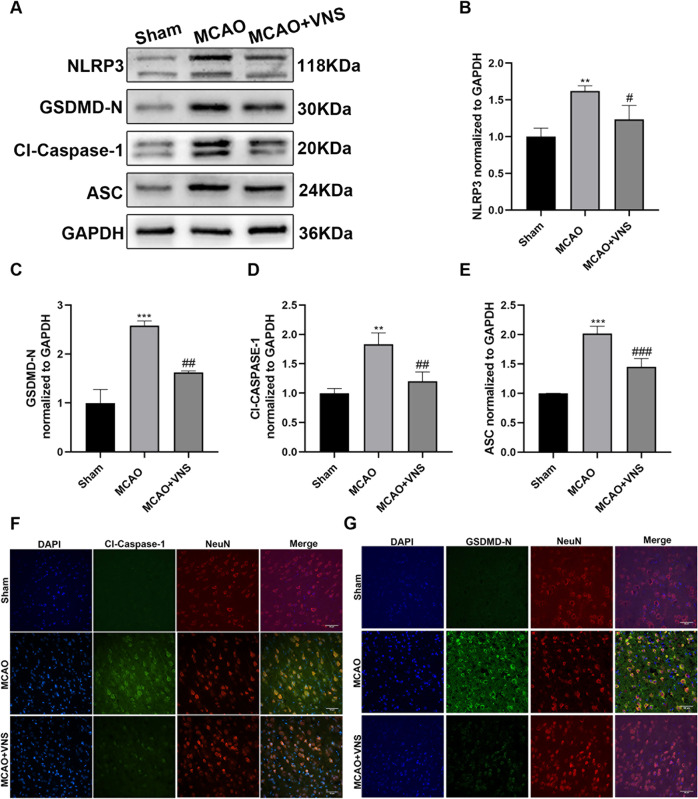
VNS suppressed the expression of NLRP3 inflammasome after cerebral ischemia/reperfusion injury. **A**–**E** Immunoblotting analysis for NLRP3, GSDMD-N, cleaved caspase 1 (Cl-Caspase-1), and ASC (*n* = 6 per group). VNS significantly downregulated the contents of NLRP3, GSDMD-N, Cl-Caspase-1, and ASC when compared to the middle cerebral artery occlusion (MCAO) group. (**F**, **G**) Representative immunofluorescence images of Cl-Caspase-1 and GSDMD-N co-stained with NeuN. VNS reduced the amounts of Cl-Caspase-1-positive and GSDMD-N-positive neurons when compared with the MCAO group. Scale bar = 50 μm. **P < 0.01, ***P < 0.001 versus the sham group and ^#^*P* < 0.05, ^##^*P* < 0.01, ^###^*P* < 0.001 versus the MCAO group.

To explore the protective roles of VNS on pyroptosis, we compared the expression of NLRP3, ASC, Cl-Caspase-1, and GSDMD-N in various groups. Western blotting results revealed that the levels of NLRP3, ASC, Cl-Caspase-1, and GSDMD-N were significantly increased after I/R injury when compared to the sham group. However, this increasing trend was reversed considerably through VNS (Fig. 2A–E). Double Immunofluorescence (IF) staining was conducted to detect the expression and location of the critical molecules of pyroptosis, namely Cl-Caspase-1 and GSDMD-N (Fig. 2F, G). IF staining results revealed that Cl-Caspase-1 and GSDMD-N were primarily co-localized with neuronal marker NeuN in the cerebral cortex. Consistent with the western blotting results, the numbers of Cl-Caspase-1-positive and GSDMD-N-positive neurons in the MCAO group significantly decreased after VNS treatment.

**Fig. 3:**
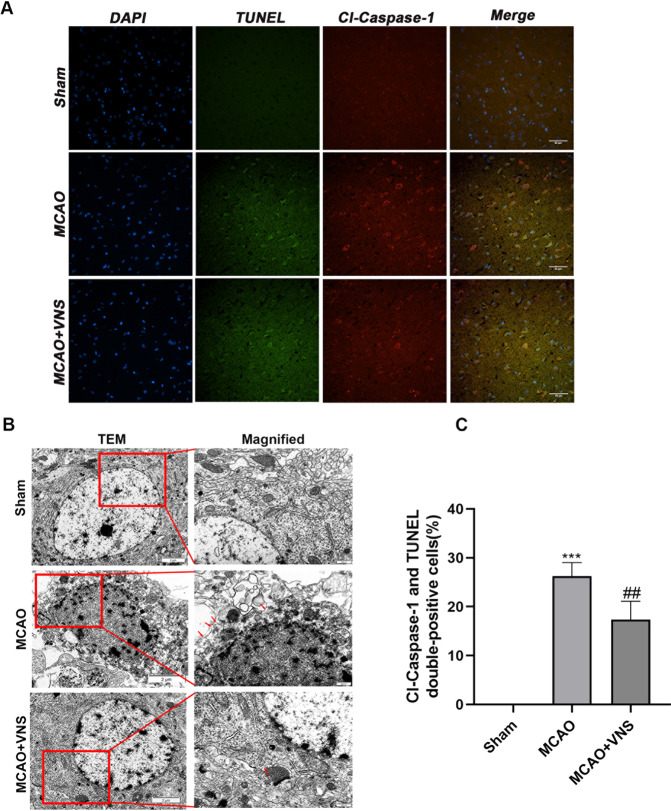
VNS suppressed cellular pyroptosis following cerebral ischemia and reperfusion injury. **A**, **C** VNS decreased the number of TUNEL (green) and Cl-Caspase-1 (red) double-positive cells when compared to the MCAO group. Scale bar = 50 μm. **B** Representative transmission electron microscopy images of neurons in the ischemic penumbra. Red boxes: magnified area, red arrowhead: membrane pores. ***P < 0.001 versus the sham group, ^##^*P* < 0.01 versus the MCAO group.

Moreover, both Cl-Caspase-1 activity and the number of TUNEL-positive cells were upregulated in the MCAO group (Fig. 3A, C), indicating that cerebral I/R injury remarkably expanded the number of pyroptotic cells. However, electrical VNS could decrease the number of pyroptotic cells. Transmission electron microscopy images revealed that neurons exhibited more membrane pores after I/R injury compared to the sham group. Nevertheless, electrical VNS downregulated the amount of membrane pores compared with the MCAO group (Fig. 3B).

### VNS reduced pyroptosis-related molecules through α7nAchR

**Fig. 4:**
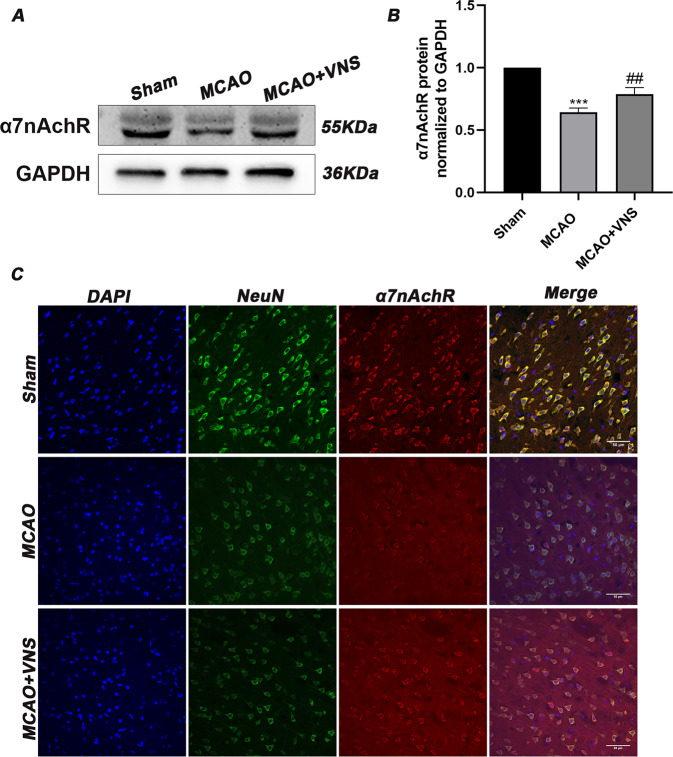
VNS upregulated the expression of α7nAchR. **A**, **B** The α7nAchR levels were decreased due to cerebral ischemia/reperfusion(I/R) injury when compared to the sham group. Nevertheless, VNS upregulated the levels of α7nAchR compared with the MCAO group (*n* = 5 per group). **C** Double immunofluorescence staining of α7nAchR and NeuN. VNS treatment corrected the reduction α7nAchR-positive neurons caused by cerebral I/R injury. Scale bar = 50 μm. ***P < 0.001 versus the sham group, ^##^*P* < 0.01 versus the MCAO group.

To validate whether VNS inhibited pyroptosis via α7nAchR, immunoblotting, and IF staining were carried out to observe the expression of α7nAchR. When comparing the sham group to the MCAO group, Western blotting revealed that I/R injury drastically reduced the contents of α7nAchR. However, when compared to the MCAO group, VNS corrected the I/R injury-induced decline in α7nAchR levels (Fig. 4A, B). Similarly, the IF staining revealed that α7nAChR-positive neurons in the VNS group were more than those in the MCAO group. In addition, α7nAChR was found to be primarily co-localized with NeuN in the cerebral cortex (Fig. 4C). To further verify the role of α7nAchR in the inhibition of pyroptosis, the corresponding antagonist MLA and agonist GTS-21 were used for downregulating or upregulating the α7nAchR levels.

**Fig. 5:**
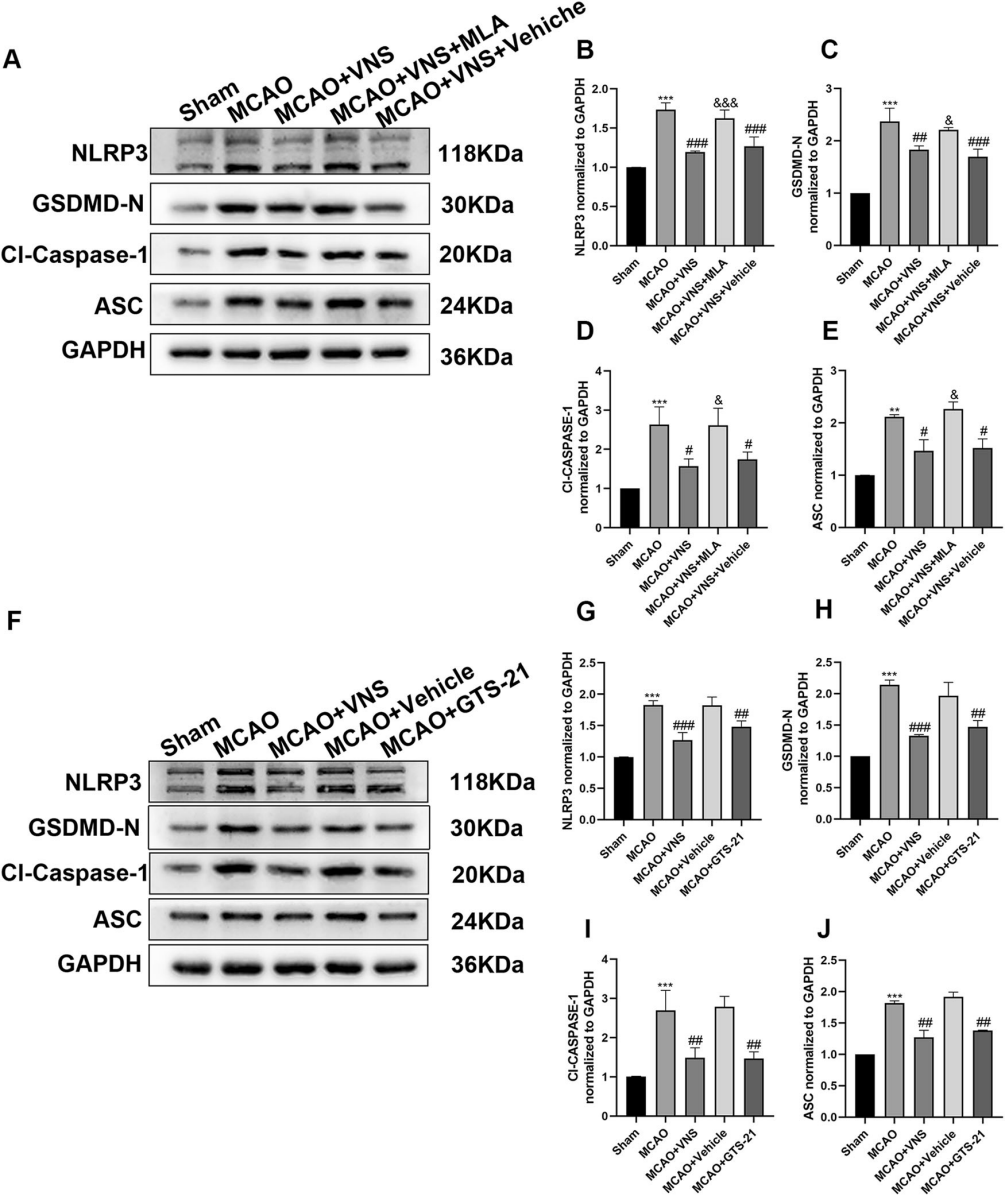
α7nAchR was involved in VNS-induced inhibition of NLRP3 inflammasome. **A–E** Representative western blotting images and analysis of NLRP3, GSDMD-N, cleaved caspase 1 (Cl-Caspase-1), and ASC (*n* = 6 per group). VNS-induced inhibition of NLRP3 inflammasome was reversed by an α7nAchR antagonist (methyl lycaconitine citrate). The levels of NLRP3, GSDMD-N, Cl-Caspase-1, and ASC were upregulated following administration of an α7nAchR antagonist when compared to the MCAO + VNS group. **F**–**J** Representative western blotting analysis of NLRP3, GSDMD-N, ASC, and Cl-Caspase-1 (*n* = 6 per group). The suppression of NLRP3 inflammasome was triggered by the α7nAchR agonist (GTS-21), which mimicked the action of VNS. When compared to the MCAO group, the NLRP3, GSDMD-N, Cl-Caspase-1, and ASC levels were decreased in the MCAO + GTS-21 group. **P < 0.01, ***P < 0.001 versus the sham group; ^#^*P* < 0.05, ^##^*P* < 0.01, ^###^*P* < 0.001 versus the MCAO group; and ^&^*P* < 0.05, ^&&&^*P* < 0.001 versus the MCAO + VNS group.

**Fig. 6:**
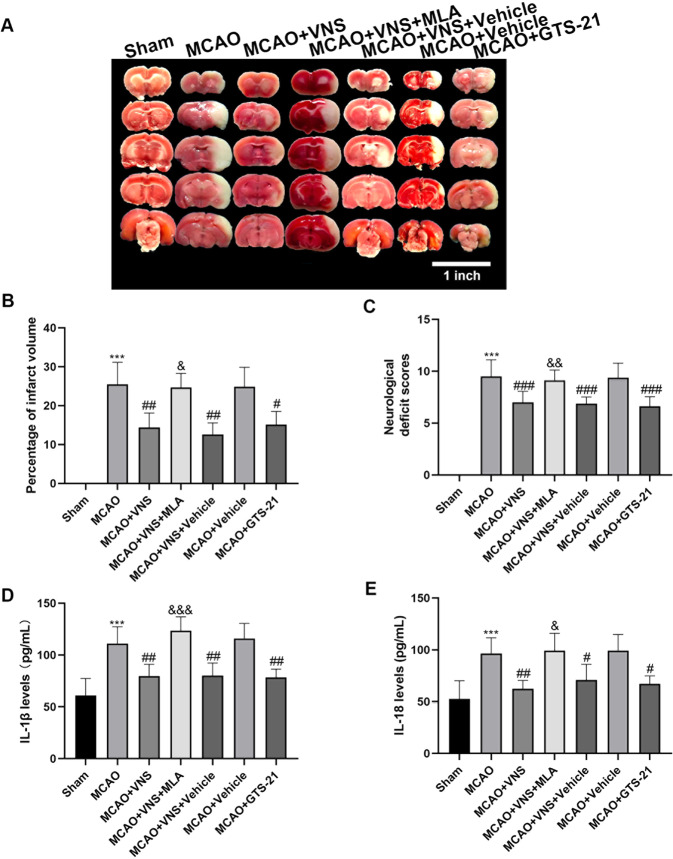
The neurological protection and anti-inflammatory effects of VNS were α7nAchR-dependent. **A**, **B** Representative 2,3,5-triphenyl tetrazolium chloride staining images at 3 days following ischemia/reperfusion injury and quantitative analysis of infarct volumes (*n* = 6 per group). **C** Assessment of the neurological scores in each group (*n* = 8 in each group). **D**, **E** Representative enzyme-linked immunosorbent assays of pyroptosis-related pro-inflammatory factors IL-1β and IL-18 (n = 6 in each group). *P < 0.05, **P < 0.01, ***P < 0.001 versus the sham group; ^#^*P* < 0.05, ^##^*P* < 0.01, ^###^*P* < 0.001 versus the MCAO group; and ^&^*P* < 0.05, ^&&^*P* < 0.01, ^&&&^*P* < 0.001 versus the MCAO + VNS group.

Western blotting results suggested that the contents of NLRP3, ASC, Cl-Caspase-1, and GSDMD-N were considerably higher in the MCAO + VNS + MLA group compared to the MCAO + VNS group (Fig. 5A–E). Intraperitoneal injection of GTS-21 significantly downregulated the expression of NLRP3, ASC, Cl-Caspase-1, and GSDMD-N compared to the MCAO group (Fig. 5F–J). When compared to the sham group, cerebral I/R injury upregulated the contents of IL-1β and IL-18, which was consistent with the western blotting results. At the same time, VNS downregulated the contents of IL-1β and IL-18 when compared to the MCAO group. The IL-1β and IL-18 expression in the MCAO + VNS + MLA group were considerably higher than in the MCAO + VNS group. However, as compared to the MCAO groups, GTS-21 downregulated the expression of IL-1β and IL-18 (Fig. 6D, E).

These findings suggest that following the cerebral I/R damage, VNS may enhance the levels of α7nAchR. MLA-induced deficit of α7nAChR reversed the inhibition of pyroptosis by VNS. GTS-21, an agonist of α7nAChR, replicated VNS’s protective action by suppressing NLRP3 inflammasome-mediated pyroptosis. In conclusion, the inhibition of pyroptosis-related molecules by VNS was α7nAChR-dependent.

### The neuroprotective effect of VNS was α7nAchR-dependent

To verify whether VNS exerted its neuroprotective effects through α7nAChR, mNSS evaluation and TTC staining were conducted, and the results were analyzed at 3 days after reperfusion (Fig. 6A–C). I/R injury significantly increased the neurological scores and infarct volumes when compared to the sham group. When compared to the MCAO group, the MCAO + VNS group performed remarkably lower neurological scores and infarct volumes. When compared to the MCAO + VNS group, the MCAO + VNS + MLA group exhibited considerably higher neurological scores and infarct volumes. The injection of GTS-21 to rats that had undergone I/R injury resulted in lower neurological scores and infarct sizes. In addition, there were no substantial differences between the MCAO + VNS group and the MCAO + VNS + vehicle group, nor between the MCAO + vehicle and the MCAO group. These findings revealed that MLA reversed VNS’s neuroprotective benefits, while GTS-21 replicated VNS’s neuroprotection. In conclusion, the neuroprotective properties of VNS were α7nAchR-dependent.

## Discussion

In the present study, we demonstrated that VNS alleviated cerebral I/R injury by suppressing NLRP3 inflammasome activation and cellular pyroptosis. Furthermore, VNS exerted neuroprotection and inhibition of pyroptosis through activation of α7nAChR and involvement of CAP. These results suggested that α7nAChR was a potential mediator of VNS-induced inhibition of pyroptosis after cerebral ischemic stroke.

As leading causes of death globally, stroke therapies have troubled clinicians for a long time [19]. Inflammation is well acknowledged to play a critical role in ischemic stroke, as evident throughout the stroke process [3, 20]. Therefore, understanding and inhibiting neuroinflammation are meaningful in the search for potential treatment. Several types of research have focused on the inflammatory mechanism involving inflammasomes after I/R injury [2]. NLRP3 inflammasome, a multi-protein complex, consists of three core proteins: NLRP3, the adaptor protein ASC, and pro-caspase 1. The NLRP3 molecule functions as an intracellular sensor for I/R injury and binds with ASC via its PYD domain. Subsequently, ASC binds and activates pro-caspase-1 [21]. Activated caspase-1 causes IL-1β and IL-18 to be cleaved and released, as well as the creation of membrane pores, which release numerous pro-inflammatory cytokines and amplify neuroinflammation [22]. In addition, pyroptosis-related pro-inflammatory cytokines including IL-1β and IL-18 have been shown in multiple studies to aggravate brain injury and inflammation [23].

Conversely, IL-1β inhibition can reduce the volume of cerebral infarcts in MCAO rats [24]. Cumulative evidence has revealed that NLRP3 inflammasome proteins and corresponding pro-inflammatory cytokines are significantly elevated in MCAO models. Consistent with these findings, the current study revealed that the contents of NLRP3 inflammasome and other principal executioners of pyroptosis were increased on day 1. This high expression was maintained until 7 days after reperfusion. The highest expression level was observed on day 3 after the I/R injury. The NLRP3 inflammasome is primarily expressed in microglia initially following cerebral I/R injury, according to Gong et al. [25], and then principally in neurons afterwards. Consistent with this observation, we noted that Cl-Caspase-1 and GSDMD-N, the principal executioners of pyroptosis, were mainly co-localized with neurons at 72 h following I/R injury. Caspase-1 expression was considerably enhanced after ischemic stroke as a critical molecule in initiating pyroptosis, and its suppression resulted in neuroprotection against I/R injury. It has been reported that vx-765, an inhibitor of caspase-1, can ameliorate blood-brain-barrier dysfunction and integrity after I/R injury [26]. GSDMD is an essential factor in pyroptosis, and its role has been reported in I/R injury. It has been well acknowledged that GSDMD-N is closely related to the formation of membrane pores and exhibits membrane-disrupting cytotoxicity [27]. Interestingly, our findings indicated that VNS downregulated the levels of GSDMD-N and reduced the number of membrane pores.

VNS is an adjunctive therapy approved by the FDA for partial epilepsy and drug-resistant depression in 1997 [28]. It is also an alternative therapy for Parkinson’s disease, Alzheimer’s disease, cluster headaches, migraines, and traumatic brain injury. It has recently been suggested that VNS offers protection against ischemic brain injury [29–31]. Our previous researches have demonstrated that VNS can attenuate cerebral I/R injury by suppressing the apoptotic response [17], inhibiting oxidative stress [32], enhancing axonal plasticity [13], and inducing angiogenesis [33]. Moreover, it has recently been reported that VNS provides its neuroprotection after I/R injury by suppressing autophagy [10]. Like autophagy, pyroptosis, a sort of programmed cell death, is important in the occurrence and progression of cerebral ischemic stroke. Interestingly, we observed that VNS improved the neuroprotective effects by inhibiting pyroptosis after cerebral I/R injury.

In clinical trials, invasive VNS paired with rehabilitation is acceptably safe and feasible in cerebral I/R patients with upper limb motor deficit [34, 35]. The similar results are shown in other studies about non-invasive VNS. Transcutaneous auricular VNS, applied noninvasively to the peripheral auricular branch of the VS, can also improve the upper limb motor function of the patients suffered from cerebral I/R injury [36–38]. Furthermore, it has been suggested that sensor function can be significantly improved in chronic stroke patients following VNS treatment paired with the conventional rehabilitation [39, 40].

The neuroprotective actions provided by VNS are mainly attributed to the anti-inflammatory properties of the VN through the release of acetylcholine following activation of α7nAChR, which is a crucial node of CAP. α7nAChR, a ligand-gated channel, is widely expressed in different areas of the CNS, such as astrocytes and microglia, but it is mainly expressed in the neurons [41]. Consistently, we observed that α7nAChR was co-localized with neurons. As the most pivotal receptor for transmitting cholinergic anti-inflammatory signals, α7nAChR plays an essential role in providing neuroprotective effects and preventing tissue injury and death in the CNS. Li et al. [15] indicated that the molecular mechanism of α7nAchR-induced anti-inflammatory effect through CAP was mainly attributed to the regulation of NF-κB transcription and activation of the JAK2/STAT3 signaling cascade to modulate the contents of pro-inflammatory cytokines. Studies have reported that VNS and α7nAChR-agonists exhibit similar positive effects on suppressing the levels of pro-inflammatory cytokines including IL-1β and tumor necrosis factor [42].

Conversely, the anti-inflammatory effect is not observed in α7nAChR-knockout mice [43]. Moreover, VNS inhibits cytokine synthesis in wild-type animals but not in mice lacking α7nAChR [44]. Our results were consistent with these findings. GTS-21, an α7nAChR-agonist, replicated the VNS-induced suppressing of IL-1β and IL-18. Nonetheless, these anti-inflammatory effects could be reversed by the administration of MLA, an α7nAChR-antagonist. Thus, VNS-induced anti-inflammatory properties in CAP are α7nAChR-dependent.

Our previous research indicates that α7nAChR is involved in inhibiting apoptosis [17] and modulating neuroinflammation [18] in the MCAO model. Reportedly, α7nAChR expression was decreased after I/R injury, and VNS could activate α7nAChR-mediated CAP to exert its neuroprotective effects [45]. Consistent with this evidence, we observed that α7nAChR levels were significantly decreased due to I/R injury, whereas VNS upregulated the expression of α7nAChR. To verify our previous hypothesis, we administered α7nAChR-agonist GTS-21 and antagonist MLA. We observed that both VNS and GTS-21 provided similar neuroprotective effects in suppressing the expression of the NLRP3 inflammasome, reducing the infarct volume, and alleviating the neurological deficits. Interestingly, these protective effects could be reversed using MLA. Our results suggested that α7nAChR and CAP were involved in the VNS-induced neuroprotective effects through inhibition of pyroptosis after cerebral I/R injury.

## Materials and methods

### Animals and experimental designs

Adult male Sprague Dawley rats weighing 240–280 g were purchased from the Experimental Animal Centre of Chongqing Medical University (Chongqing, China). All rats were kept in a 12-h light/dark cycle room maintained at a temperature of 21–23 °C and relative humidity of 60%. They were permitted to consume food and water ad libitum throughout the study period. All experiments were performed following the National Institute of Health guidelines for the care and use of animals. All animal procedures were approved by the Ethics Committee of Chongqing Medical University. The whole study was divided into three experiments and all rats were divided into the different groups by using the random number table method.

#### Experiment 1

To detect the time course of NLRP3 expression after I/R injury in rats, they were randomly divided into five groups (*n* = 8 per group): (1) sham group, (2) 6-h MCAO group, (3) 1-day MCAO group, (4) 3-day MCAO group, and (5) 7-day MCAO group.

#### Experiment 2

To investigate whether electrical VNS can attenuate cerebral I/R injury in rats through anti-pyroptosis pathways, they were randomly assigned to three groups (*n* = 8 per group): (1) sham group, (2) MCAO group, and (3) MCAO + VNS group.

#### Experiment 3

To verify whether VNS can inhibit pyroptosis through activation of α7nAChR in rats, they were randomly divided into seven groups (n = 7 per group): (1) sham group, (2) MCAO group, (3) MCAO + VNS group, (4) MCAO + VNS + α7nAChR antagonist methyl lycaconitine citrate (MLA) group, (5) MCAO + VNS + vehicle group, (6) I/R + vehicle group, and (7) MCAO + α7nAchR agonist GTS-21 group.

### Middle cerebral artery occlusion model and VNS treatment

I/R injury was established by MCAO/reperfusion as described previously [5, 46]. Briefly, rats were deeply anesthetized using 3.5% chloral hydrate, and the airway was kept unobstructed. The right common carotid artery, external carotid artery, and internal carotid artery were exposed via a midline neck incision. A nylon suture was inserted and advanced through the carotid bifurcation to block the origin of the middle cerebral artery for 120 min. The suture was released after 120 min of occlusion, followed by reperfusion.

The same operation was applied to the rats in the sham group but without the insertion of the suture. The body temperature of the rats was kept approximately at 37 °C through a heating lamp for the duration of the surgery. Heart rate (HR), blood gas concentrations, and tail arterial pressure were recorded.

Invasive VNS was executed as described in previous studies [47, 48]. Briefly, the nerve stimulation electrodes were placed nearly the right cervical VN and sutured at this position. The rats in VNS group underwent nerve stimulation for 1 h at 30 min after MCAO. The stimulation parameters used were 30 sec ON and 5 min OFF, with each ON period consisting of 0.5 msec pulses delivered at 0.5 mA,20 Hz.

### Administration of α7nAchR-agonist and antagonist

To explore its role in the anti-pyroptosis pathway, we selectively inhibited or activated α7nAChR. According to our research, the corresponding antagonist MLA (6 mg/kg; MCE, USA) and agonist GTS-21 (4 mg/kg; MCE, USA) were used [13, 14]. As described in a previous study, we dissolved MLA and GTS-21 in phosphate-buffered saline (PBS) and delivered them intraperitoneally into the rats 30 min before VNS [14]. In the corresponding control group, we ensured that the volume of PBS was consistent with the dose of the antagonist or the agonist.

### Evaluation of neurological function

The functional deficit was evaluated by a blinded observer using the modified neurological severity score (mNSS) on day 3 after MCAO and reperfusion. The mNSS is an 18-point neurobehavioral scoring system (from no deficit [a score of 0] to maximal deficit [a score of 18]). It includes motor function test, sensory test, beam balance test, reflex absence, and tests for abnormal movements [49, 50]. In the trial, if a corresponding neurological deficit exists, the related scores are added. Therefore, higher scores indicate a more severe neurological deficit.

### Measurement of infarct volume

Infarct volume measurement was carried out on day 3 after I/R injury as described previously [51]. Briefly, after the rats were deeply anesthetized, brains were quickly removed and evenly chopped into 2-mm-thick slices. Subsequently, five slices were immersed entirely into 2% 2,3,5-triphenyl tetrazolium chloride (TTC) at 37.5 °C for 25 min, followed by soaking in 4% paraformaldehyde for 12 h. Finally, these brain slices were imaged with a camera and the infarct volumes were analyzed and calculated using the ImageJ software.

### Western blot analysis

Protein samples from the brain tissues were lysed in radioimmunoprecipitation assay buffer (Beyotime, Nantong, China) with 1 mM protease inhibitor (Boster, Wuhan, China). Protein concentrations were determined using a BCA protein assay kit (Beyotime, Nantong, China) was used to quantify Protein concentrations. Western blotting was conducted as previously reported [52]. Membranes were blocked with 8% non-fat dried milk at room temperature for 1 h, then incubated with primary antibodies GSDMDC1 (1:200, Santa Cruz, sc-393656), α7nAChR (1:100, Santa Cruz, sc-58607), ASC (1:200, Santa Cruz, sc-514414), NLRP3 (1:1000, Abcam, ab263899), caspase1 (1:1000, Proteintech, 22915-1-AP), GAPDH (1:5000, Proteintech, 10494-1-AP), and β-tubulin (1:5000, Proteintech, 10094-1-AP) overnight at 4 °C. After incubating the membranes with corresponding secondary antibodies, images were captured and quantified using the Fusion FX5 analysis system and analyzed using the Quantity One software.

### Immunofluorescence and TUNEL staining

IF staining was performed on 16-μm-thick frozen brain sections as described previously [53]. Briefly, the brain sections were permeabilized with 0.35% Triton X-100 for 15 min, blocked for 60 min at 37 °C with goat serum, then incubated with the matching primary antibodies at 4 °C overnight. The primary antibodies included mouse anti-caspase 1 (1:40, Santa Cruz, sc-56036), mouse anti-NeuN (1:200, MilliporeSigma, MAB377), mouse anti-GSDMDC1 (1:50, Santa Cruz, sc-393656), rabbit anti-NeuN (1:100, Proteintech, 26975-1-AP), and rabbit anti-α7nAChR (1:50, Bioss, bs-1049R). On the following day, the slides were incubated with corresponding secondary antibodies and 4′,6-diamidino-2-phenylindole. Subsequently, the slides were imaged using a microscope (Nikon, Tokyo, Japan) and analyzed using the ImageJ software.

TUNEL staining (Boster, Wuhan, China) was used to count the number of dead cells, following the manufacturer’s instructions as previously described [54]. The number of TUNEL-positive cells was statistically analyzed by a blinded observer using the ImageJ software.

### Transmission electron microscopy

Under deep anesthesia, 0.1 M PBS and 2.5% ice-cold glutaraldehyde were used to perfuse the rats. Subsequently, brain tissues from the ipsilateral cortex were cut into 1-mm³ sections, which were then soaked in 2.5% glutaraldehyde and followed fixed in 1% osmium tetroxide [55]. Finally, uranyl acetate and lead citrate were used to stain the samples which ultimately were scanned using an H7500 transmission electron microscope (Hitachi, Tokyo, Japan).

### Enzyme-linked immunosorbent assay

Cerebral cortex tissues were taken to determine the contents of IL-1β and IL-18 at 3 days after I/R injury. The expression levels of IL-1β and IL-18 in the tissue homogenate were tested with their corresponding kits, as directed by the manufacturer.

### Statistical analysis

The data were collected by the blinder and presented as means ± standard deviations. GraphPad Prism 8.0 (GraphPad, San Diego, CA, USA) was used for constructing the graphs. For statistical analysis, IBM SPSS Statistics 25.0 (IBM Corp., Armonk, NY, USA) was utilized. The differences between the groups were analyzed using one-way or two-way analysis of variance followed by Tukey’s post hoc multiple comparison test. The threshold for statistical significance was fixed at *P* *<* 0.05.

## Data Availability

The data used to support the findings of this study are included in the paper article.
